# Comparative Study of Cytotoxicity and Antioxidant, Anti-Aging and Antibacterial Properties of Unfermented and Fermented Extract of *Cornus mas* L.

**DOI:** 10.3390/ijms241713232

**Published:** 2023-08-26

**Authors:** Martyna Zagórska-Dziok, Aleksandra Ziemlewska, Agnieszka Mokrzyńska, Zofia Nizioł-Łukaszewska, Ireneusz Sowa, Dariusz Szczepanek, Magdalena Wójciak

**Affiliations:** 1 Department of Technology of Cosmetic and Pharmaceutical Products, Medical College, University of Information Technology and Management in Rzeszow, Sucharskiego 2, 35-225 Rzeszow, Poland; mzagorska@wsiz.edu.pl (M.Z.-D.); aziemlewska@wsiz.edu.pl (A.Z.); amokrzynska@wsiz.edu.pl (A.M.); zniziol@wsiz.edu.pl (Z.N.-Ł.); 2 Department of Analytical Chemistry, Medical University of Lublin, Aleje Raclawickie 1, 20-059 Lublin, Poland; i.sowa@umlub.pl; 3 Chair and Department of Neurosurgery and Pediatric Neurosurgery, Medical University of Lublin, Jaczewskiego 8, 20-090 Lublin, Poland; dariusz.szczepanek@umlub.pl

**Keywords:** *Cornus mas* L., antioxidants, kombucha, skin cells, anti-aging activity, antibacterial activity

## Abstract

Due to the high demand for products that can help treat various skin conditions, the interest in plant extracts, which are a valuable source of phytochemicals, is constantly growing. In this work, the properties of extracts and ferments from *Cornus mas* L. and their potential use in cosmetic products were compared. For this purpose, their composition, antioxidant properties and cytotoxicity against skin cells, keratinocytes and fibroblasts were assessed in vitro. In addition, the ability to inhibit the activity of collagenase and elastase was compared, which enabled the assessment of their potential to inhibit skin aging. Microbiological analyses carried out on different bacterial strains were made in order to compare their antibacterial properties. The conducted analyses showed that both dogwood extract and ferment have antioxidant and anti-aging properties. In addition, they can have a positive effect on the viability of keratinocytes and fibroblasts and inhibit the proliferation of various pathogenic bacteria, which indicates their great potential as ingredients in skin care preparations. The stronger activity of the ferment compared to the extract indicates the legitimacy of carrying out the fermentation process of plant raw materials using kombucha in order to obtain valuable products for the cosmetics industry.

## 1. Introduction

Plants have always been considered an extremely valuable source of chemical compounds with versatile effects [1]. Hence, extracts, decoctions or infusions have been used for centuries in the treatment and elimination of the adverse effects of many types of diseases [2]. Nowadays, plant extracts are more and more often used in the food, pharmaceutical and cosmetic industries, mainly due to the wide range of bioactive compounds such as carotenoids, glycosides, saponins, flavonoids, iridoids, alkaloids, essential oils, tannins, organic acids or vitamins [3,4]. Despite this, new products of plant origin are constantly being sought, which would be a rich source of new compounds with broad biological activity. One of the extremely popular ways of obtaining such products is to carry out the fermentation process, which leads to obtaining additional compounds that are not obtained during the extraction of individual plant raw materials [5].

*Cornus mas* L., also known as dogwood, is a plant of the *Cornaceae* family that grows in Eastern Europe and the Middle East. It is one of the most valuable fruit plants that belong to this family [6]. The popularity of dogwood fruit is constantly growing. Dogwood is eaten in dried, pickled form, but also as jams, marmalades, juices and in fresh form [7]. Dogwood is cultivated for its ornamental value, but also for its many health benefits [8]. In recent years, there has been increasing interest in studying the biological properties of dogwood and its potential use in the field of medicine and health. Research focuses on the analysis of bioactive ingredients and their impact on the human body and the possibility of using this plant in the treatment of various diseases [9]. Literature reports indicate that dogwood, in addition to having a high content of vitamin C, is a rich source of many biologically active compounds such as anthocyanins, phenolic acids, flavonoids, organic acids and iridoids [10]. The compounds contained in dogwood fruits cause dogwood to have antioxidant, anti-inflammatory, antimicrobial, antiproliferative, antiatherosclerotic, antidiabetic and immunomodulatory properties [11,12,13].

Dogwood berries are characterized by a high content of antioxidant compounds, such as vitamin C or polyphenolic compounds [14,15]. Among the anthocyanins found in dogwood fruits are: 3-O-galactosides of delphinidin, cyanidin-3-O-galactoside and 3-O-rutinoside [16]. It is also worth mentioning the presence of flavonoids and phenolic acids, such as: caffeic acid, ellagic acid, chlorogenic acid, gallic acid, quercetin and catechin derivatives [6]. In addition, iridoids such as cornuside and loganic acid are present in *Cornus mas* L., which are characterized by anti-inflammatory and antioxidant properties [16,17]. The hydroxybenzoic acids present in dogwood fruits are gallic acid and ellagic acid [18]. Ellagic acid has immunostimulating, immunomodulating, antimicrobial, antioxidant, anti-inflammatory and anticancer effects, and also inhibits the adverse effects of UVA and UVB radiation on the skin [19,20]. In addition, dogwood is a rich source of minerals such as calcium, potassium, phosphorus, sodium, magnesium, iron, zinc and copper [21]. The composition of biologically active substances varies depending on the part of the plant [21,22].

In addition to the fact that dogwood is used for food purposes, it is a plant with potential skin care properties. The potential use of dogwood in cosmetics can help improve skin condition, alleviate inflammation, reduce discoloration and protect against oxidative stress [11,23]. Oxidative stress is an imbalance between reactive oxygen species and the body’s ability to neutralize them through antioxidant mechanisms [24]. Research is being conducted around the world on the role of oxidative stress in skin aging. Current knowledge indicates that the increase in ROS (reactive oxygen species) leads to chronic inflammation, which may result in collagen fragmentation and disorganization of collagen fibers. In addition, oxidative stress causes the skin to lose its natural functional characteristics and regenerative potential. Moreover, the skin undergoes functional senescence, which is primarily associated with a reduced ability to counteract various infections, which promotes the occurrence of various types of autoimmune and cancer diseases [11,25,26].

Although extracts obtained from various plants are undeniably a huge source of compounds with a wide biological activity, new raw materials and products are constantly being sought that will be able to support the treatment of various diseases, including skin diseases [27]. The constant improvement of extraction methods and the selection of appropriate solvents contribute to obtaining more and more valuable products, but difficulties in the treatment of some diseases require a constant search for new compounds or products with desired properties in the context of treating various diseases [28]. Due to the wide biological activity and the possibility of obtaining large amounts of various chemical compounds, products obtained by fermentation of plant materials are becoming more and more popular [29]. As numerous scientific reports show, the fermentation of various plant extracts results in obtaining ferments with extremely valuable properties in the context of caring for the healthy appearance of the skin and eliminating skin changes occurring in the case of various diseases of this organ. According to literature reports, the juice of *C. mas* L. is considered as an alternative fermentation substrate for the production of fermented beverages. In the juice and unripe fruit of dogwood fermented by *Lactobacillus* sp., a simultaneous decrease in total sugars and an increase in lactic and acetic acid content was observed [30]. Moreover, it was shown that the fermented extract of *C. mas* L. had a significant content of iridoids and phenolic acids in its composition, which may have a beneficial effect on the gut and skin microbiota and, thus, play a role as new probiotic functional products [31].

A fermentation method that allows obtaining versatile ferments is the use of a symbiotic culture of bacteria and yeast called a SCOBY [32]. Literature reports indicate the extraordinary pharmacological activity of kombucha products obtained during the fermentation of various types of raw materials, including, among others, antioxidant, anti-inflammatory, anti-aging, immunomodulating, anti-cancer, anti-microbial, anti-diabetic, anti-hypertensive and anti-hyperlipidemic effects [33,34]. Hence, the interest in fermented plant extracts is constantly growing due to the high hopes associated with the use of these ferments in the treatment and elimination of changes occurring during various types of skin disorders.

The main objective of these studies was to compare the biological activity of extract and ferment obtained from *Cornus mas* L. and to assess the possibility of their use in products intended for the care and treatment of skin diseases. For this purpose, the presence of chemical compounds in the extract and ferment was compared, and the antioxidant, anti-aging, promigratory, antibacterial and cytotoxic properties of the obtained samples were assessed.

## 2. Results and Discussion

### 2.1. Phytochemical Analysis 

**Figure 1:**
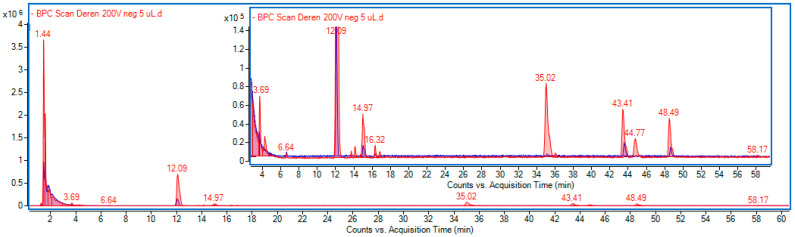
Overlapped BPC chromatograms *C. mas* L. extract (blue line) and fermented extract (red line) obtained in negative ionization mode.

The components of water extract and fermented extract from the fruits of *Cornus mas* L. were separated using reversed-phase chromatography. The identification of the compounds was carried out based on the acquisition of mass data obtained in the negative ionization mode (m/z-H) and UV-Vis spectra (200–600 nm), and comparison with standards when available or with data given in the literature. Representative chromatograms are shown in Figure 1. 

Two groups of compounds were predominant in the analyzed extracts, i.e., polyphenols and iridoids. Among the polyphenols, gallic acid and gallotannins, namely, galloyl-d-sedoheptulose and O-galloyl-d-glucose, were the most abundant, and their concentrations were increased in the fermented extract. They displayed a common MS pattern characteristic for galloyl derivates with m/z 169 (gallic acid-H) and m/z 125 (decarboxylated galloyl). A low concentration of protocatechuic acid, dihydroxybenzoic acid glucoside, and cyanidin 3-O-galactoside, belonging to anthocyanins, was also found in both extracts. Meanwhile, chlorogenic acid was only present in the fermented extract. 

Loganic acid and its derivatives, as well as secoxyloganin and cornuside, were the main iridoids found in the extracts, which is in line with the literature data [35,36]. Among them, loganic acid was the predominant component followed by secoxyloganin and cornuside. The content of iridoids was significantly higher in fermented extract. Furthermore, three prominent peaks were observed in the region of 43–49 min that did not exhibit absorption in the range of 200–600 nm. They have not been identified, and their formulas were generated by MassHunter software (ver. 10.0). The registered mass spectra are presented in the Supplementary material (Figure S1). These components significantly increased as a result of fermentation. 

Mass data and results of quantitative analysis are summarized in Table 1. MS spectra for main identified compounds in *C. mas* L. extract are presented in Figure S2 (Supplementary material).

### 2.2. Assessment of Antioxidant Activity

#### 2.2.1. DPPH and ABTS^•+^ Radical Scavenging

To assess the antioxidant potential of the tested extract and ferment, DPPH, ABTS and Fe^2+^ chelation assays were used. The first two are widely used methods to assess the antioxidant properties of various types of natural products. These are techniques using spectrophotometric measurements based on free radical quenching analysis. The antioxidant activity of the dogwood extract and ferment was expressed by the value of the IC50 parameter, which determines the concentration of the tested sample causing a 50% decrease in the initial concentration of the radical used (DPPH or ABTS). The antioxidant properties of the *C. mas* L. extract and ferment were analyzed over a concentration range of 1 to 5000 µg/mL (Table 2). The obtained results indicated concentration-dependent scavenging of free radicals in both assays. These results indicate that both the extract and the ferment show similar antioxidant properties, with no statistically significant differences (for the ABTS assay) and small statistically significant differences (for the DPPH assay) indicating a slightly stronger antioxidant potential of the ferment (Table 2).

**Figure 2:**
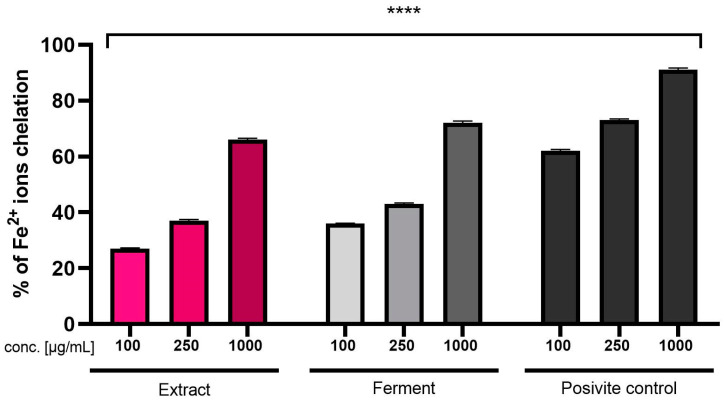
Fe^2+^ chelation by *Cornus mas* L. extract and ferment. Data are mean ± SD of three independent experiments, each consisting of three replicates. **** *p* < 0.0001 compared to negative control. Negative control: ethanol solution (1000 μg/mL). Positive control: ascorbic acid.

Due to the fact that Fe^2+^ ions are involved in the formation of reactive oxygen species that can cause oxidative stress, the next test assessing the antioxidant properties of the tested extract and ferment was the assay assessing the chelating ability of these ions (Figure 2). The obtained results indicate dose-dependent chelation of Fe^2+^ ions. At a concentration of 1000 μg/mL, a slightly higher chelating capacity of these ions was noted for the ferment (72.17%) than for the extract (66.34%), which confirms the stronger antioxidant properties of the fermented extract shown in the two assays described above. The effect is analogous for the lower of the concentrations used. 

As indicated by literature data, *C. mas* L. fruit extract contains four classes of biologically active compounds, namely, iridoids, anthocyanins, phenolic acids and flavonols [37]. The antioxidant properties of plant extracts are correlated with the total content of polyphenols and flavonoids, among which anthocyanins have strong properties [38]. Detailed phytochemical analysis has shown that, among other things, high concentrations of loganic and gallic acid and their derivatives may be responsible for the free radical scavenging effect [39,40]. In addition to polyphenols, ascorbic acid and carotenoids in *C. mas* L. play a significant antioxidant role [41]. What is more, Seeram et al. have shown that cyanidin 3-O-galactoside and pelargonidin 3-O-galactoside detected in *C. mas* L. extract inhibited lipid peroxidation by 70.2 and 40.3%, respectively, in an iron-catalysed liposomal model [42]. On the other hand, the antioxidant properties, including protection of catalase and glutathione peroxidase, of extract are also attributed to polysaccharides [43]. Furthermore, fermented plant extracts have a documented high content of active substances acting as antioxidants [44].

#### 2.2.2. Intracellular ROS Levels in Skin Cells

**Figure 3:**
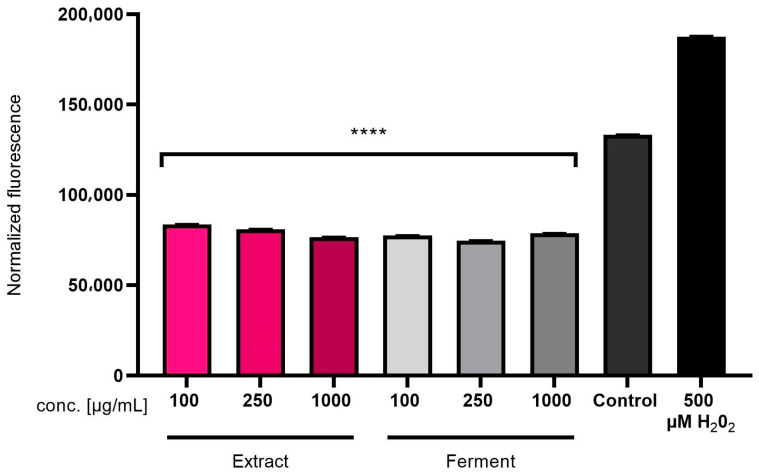
Effect of *C. mas* L. extract and ferment on DCF fluorescence in HaCaT cells. Data are the mean ± SD of three independent experiments, each consisting of three replicates per treatment group. **** *p* < 0.0001 compared to the positive control (cells treated with 500 µM H_2_O_2_).

**Figure 4:**
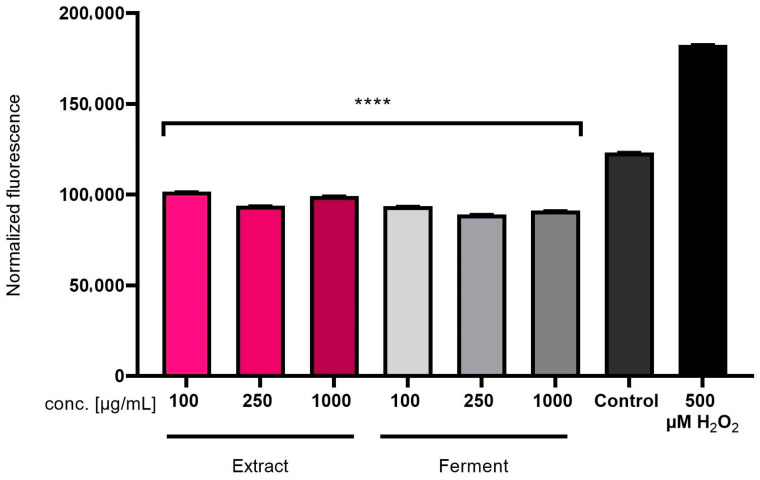
Effect of *C. mas* L. extract and ferment on DCF fluorescence in BJ cells. Data are the mean ± SD of three independent experiments, each consisting of three replicates per treatment group. **** *p* < 0.0001 compared to the positive control (cells treated with 500 µM H_2_O_2_).

In order to assess the protective effect of the *C. mas* L. ferment and extract on skin cells in vitro, the level of reactive oxygen species (ROS) in cells subjected to oxidative stress caused by treatment with hydrogen peroxide was assessed. The obtained results showed that both the extract and the ferment showed antioxidant activity and reduced the level of ROS caused by the action of H_2_O_2_. This effect was observed in both keratinocytes (HaCaT) (Figure 3) and fibroblasts (BJ) (Figure 4). Moreover, the tested samples at all analyzed concentrations were able to reduce the level of free radicals in cells even below the level of ROS in control cells (not previously treated with H_2_O_2_). The analyses carried out using the H_2_DCFDA (2′,7′-dichlorodihydrofluorescein diacetate) fluorescent probe showed stronger antioxidant properties of the ferment than the *C.mas* L. extract, which is most likely due to the higher content of biologically active compounds with antioxidant properties.

### 2.3. Cytotoxicity Assessment

**Figure 5:**
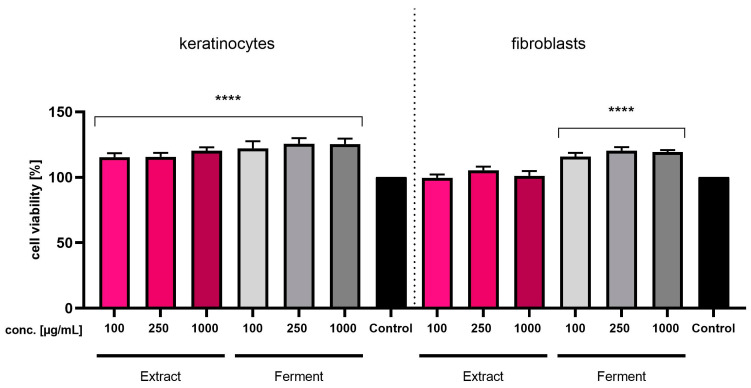
Assessment of resazurin reduction by keratinocytes (HaCaT) and fibroblasts (BJ) after 24-h exposure to *Cornus mas* L. extract and ferment (at concentrations of 100, 250 and 1000 µg/mL). Data are the mean ± SD of three independent experiments, each of which consists of three replicates per treatment group. **** *p* < 0.0001 compared with the control (100%).

In the next stage of the study, the cytotoxic properties of *Cornus mas* L. extract and ferment were evaluated. The analyses were carried out on two cell lines—fibroblasts (BJ) and keratinocytes (HaCaT). The tests used in the study (Neutral Red and Alamar Blue) made it possible to assess the degree of growth inhibition depending on the concentration of the tested samples. At the beginning, the Alamar Blue assay was performed, in which resazurin is used as an oxidation–reduction indicator, which undergoes a colorimetric change as a result of changes in cell metabolism, which enables the assessment of cell viability in vitro. Figure 5 shows the results of the analysis performed with the Alamar Blue test. Analyses performed on keratinocytes showed that the values of cell viability were significantly higher compared to the control in all analyzed samples, i.e., for all concentrations of both the extract and the ferment. In the case of fibroblasts, the ferments at all concentrations used showed significantly higher cell viability values, while the values obtained for the extracts at each concentration were at levels comparable to the control (cells not treated with the tested samples).

**Figure 6:**
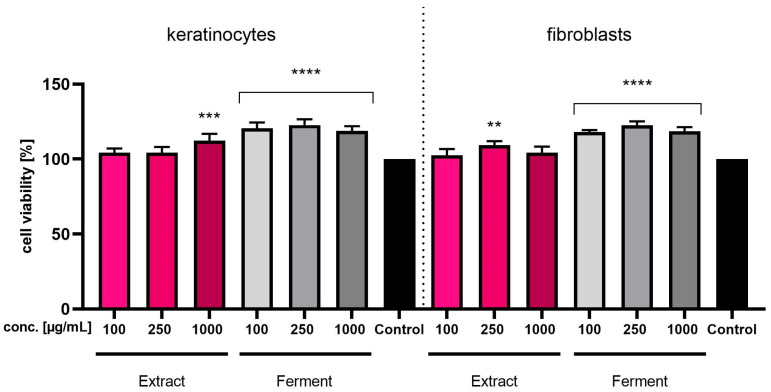
Assessment of Neutral Red Dye uptake by keratinocytes (HaCaT) and fibroblasts (BJ) after 24-h exposure to *Cornus mas* L. extract and ferment (at concentrations of 100, 250 and 1000 µg/mL). Data are the mean ± SD of three independent experiments, each of which consists of three replicates per treatment group. **** *p* < 0.0001, *** *p* < 0.003, ** *p* < 0.006 compared to the control (100%).

In order to assess the cytotoxicity of the tested samples on skin cells, additional analyses were performed using Neutral Red assay. By measuring the amount of dye released, this assay determined the total number of viable cells treated with various concentrations of *C. mas* L. extract and ferment. The results of the Neutral Red test indicate that none of the concentrations used, either of the extract or of the ferment, has a cytotoxic effect on both types of skin cells in vitro. In the case of extracts, some of the concentrations used stimulate the viability of both types of cells, while in the case of ferments, all tested concentrations cause a statistically significant increase in the viability of the tested cells (Figure 6). The lack of cytotoxicity of the tested samples during the conducted analyses suggests the potential possibility of using both the dogwood extract and the ferment in preparations applied to the skin. Moreover, the protective effect shown in the analyses assessing the intracellular level of ROS in BJ and HaCaT cells suggests a protective effect of the tested samples against pro-oxidative factors.

**Figure 7:**
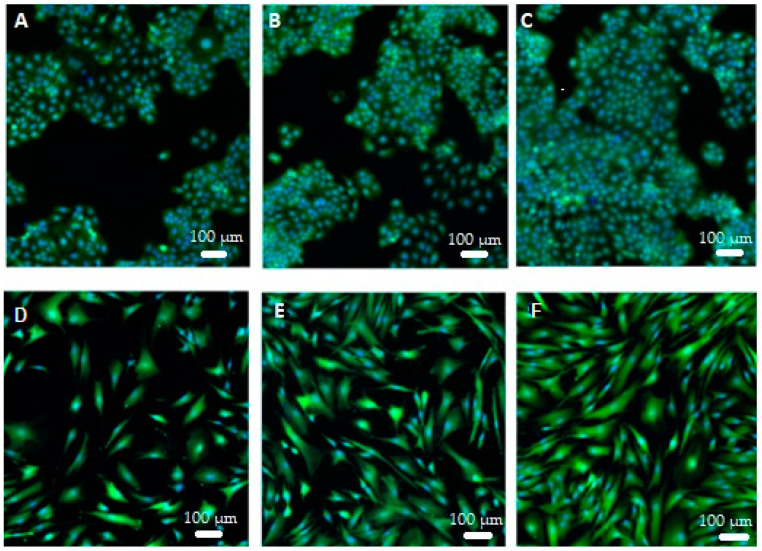
Morphology of keratinocytes (upper panel) and fibroblasts (lower panel) stained with Hoechst33342 and calcein AM (scale bar: 100 µm). The pictures were taken with an inverted fluorescence microscope and show control keratinocytes (**A**), keratinocytes treated with extract (1000 µg/mL) (**B**), keratinocytes incubated with ferment (1000 µg/mL) (**C**), control fibroblasts (**D**), fibroblasts treated with extract (1000 µg/mL) (**E**) and fibroblasts incubated with ferment (1000 µg/mL) (**F**).

In addition, microscopic images of keratinocytes (HaCaT) and fibroblasts (BJ) made using an inverted fluorescence microscope showed that both the extract and the bioferment did not adversely affect the morphology of skin cells in vitro. Moreover, the photos show enhanced proliferation of both cell types treated with both *Cornus mas* L. extract and ferment at a concentration of 1000 µg/mL compared to control cells (Figure 7).

In conclusion, it has been shown that the active substances contained in dogwood fruits can have a beneficial effect on our skin, successfully replacing many synthetic substances used in cosmetic products in the future. The beneficial effect on the proliferation and viability of skin cells shown for both extracts and ferments is probably primarily due to the presence of biologically active compounds contained in *C. mas* L., which have a positive effect on these cells by limiting the intracellular level of free radicals [45]. The more beneficial effect on skin cells caused by the ferment may be closely related to its antioxidant effect, which, in addition to the increased content of phytochemicals, may also be due to the action of antioxidant polysaccharides and peptides produced during the microbial biotransformation of the extract. In addition, the fermentation of plant materials may promote the disintegration of their cell walls, which may result in the release or production of various compounds with cytoprotective and antioxidant effects [46]. Moreover, the fermentation process can result in the release of polyphenols from complexes with antinutrients, which significantly increases the bioavailability of these biologically active compounds [47].

### 2.4. Assessment of Matrix Metallopeptidase Inhibition

Aging is a complex, multistep process that includes, among other things, the breakdown of collagen and elastin fibers [48]. Collagen and elastin are proteins that form the majority of the extracellular matrix in the skin. Collagen is responsible for the tensile strength of the skin, while elastin fibers give it the ability to stretch. These proteins, therefore, provide the skin with its normal strength, hydration and mechanical properties. These proteins provide the skin adequate strength and hydration, and also affect its mechanical properties. [49]. The enzymes elastase and collagenase are metalloproteinases that can degrade molecules such as aggrecan, fibronectin, gelatin and laminin, as well as elastin and collagen. Their increased activity leads to degenerative changes in the skin, which are visible in the form of wrinkles [50]. Therefore, agents that inhibit elastase and collagenase activity may have a beneficial effect on maintaining healthy skin by preventing degradation of the dermal matrix [51].

**Figure 8:**
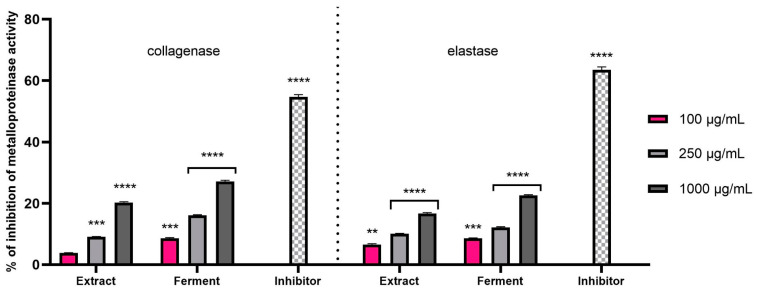
Assessment of inhibition of collagenase and elastase activity by *C. mas* L. extract and ferment at concentrations of 100, 250 and 1000 μg/mL. Data are the mean of three independent experiments, each consisting of two replicates per treatment group. **** *p* < 0.0001, *** *p* < 0.0004, ** *p* < 0.0021 comparing to the negative control.

In this study, the inhibitory effects of kombucha extract and ferment on the aforementioned enzymes were investigated to elucidate their therapeutic potential for anti-wrinkle effects. As shown in Figure 8, the tests were carried out with three concentrations of the test samples (100, 250 and 1000 ug/mL). In the case of both tested enzymes, statistically significant inhibitory effects of both the extract and the ferment were observed. Enzyme inhibition increased with increasing concentration of the tested samples, but in the case of ferment, this effect was stronger. Although the inhibition of the activity of the tested enzymes by the *C.mas* L. extract and ferment was lower than the effect of commonly known inhibitors of these enzymes, these results indicate the possibility of delaying, to some extent, the skin aging processes and the degradation of elastin and collagen fibers by the tested samples.

The anti-aging activity confirmed in the study above that extract and ferment is due to the content of biologically active compounds with proven anti-aging activity [52], such as gluconic acid, gallic acid and chlorogenic acid which is also confirmed by chromatographic analysis. Moreover, loganic acid and its derivatives, as well as catalpol as iridoid glycosides present in *Cornus mas* L., are valuable anti-inflammatory or antiaging agents [53,54]. The possibility of inhibiting the activity of enzymes responsible for skin aging by dogwood extract and ferment, shown for the first time in this work, indicates that this raw material can be perceived as a source of anti-aging phytochemicals. Due to the fact that this effect is probably the result of the interaction of biologically active compounds contained in the dogwood extract and ferment, the improvement of the method of extraction and biofermentation may contribute to obtaining dogwood samples with a much greater potential for inhibiting the aforementioned matrix metalloproteinases.

### 2.5. Wound Scratch Assay

**Figure 9:**
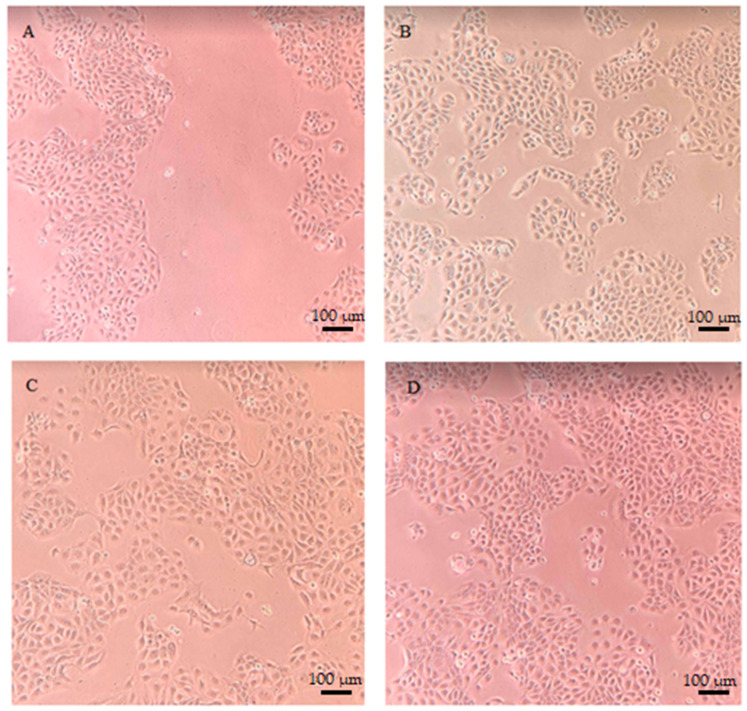
Effect of *Cornus mas* L. extract and ferment (at a concentration of 1000 µg/mL) on the migration of HaCaT cells after 24-h incubation. The pictures show in sequence: a scratch made with a pipette tip (**A**), control cells (**B**), cells treated with the extract (**C**) and cells treated with ferment (**D**). The photo was taken under an inverted microscope at × 10 magnification (scale bar: 100 µm).

**Figure 10:**
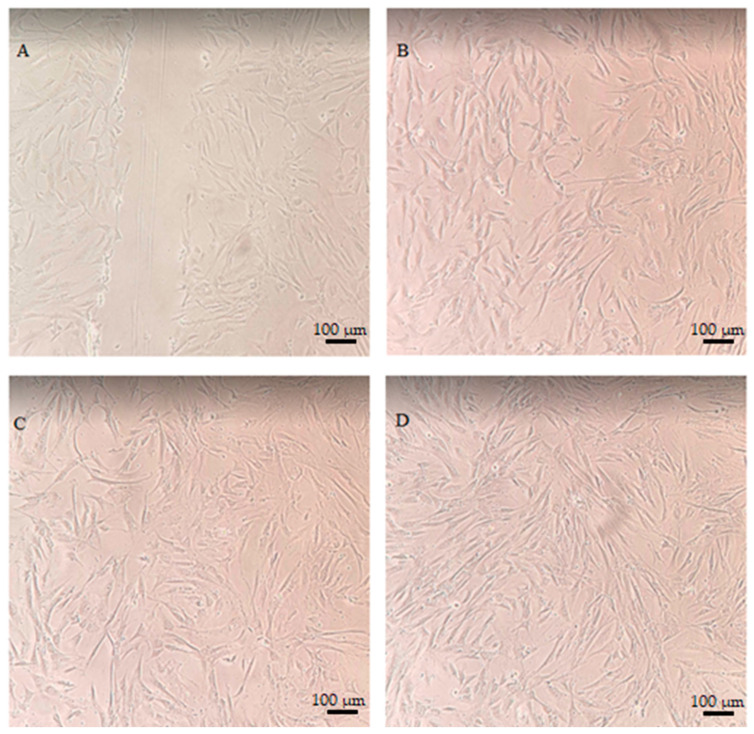
Effect of *Cornus mas* L. extract and ferment (at a concentration of 1000 µg/mL) on the migration of BJ cells after 24-h incubation. The pictures show in sequence: a scratch made with a pipette tip (**A**), control cells (**B**), cells treated with the extract (**C**) and cells treated with ferment (**D**). The photo was taken under an inverted microscope at × 10 magnification (scale bar: 100 µm).

As part of the study, a scratch test was carried out, which allows the evaluation of the measurement of the rate of migration of the examined cells [55]. This test can be used to analyze the migration of cells of various cell types, including keratinocytes or skin fibroblasts, which exhibit collective migration, also known as “sheet migration” [56]. Cell pictures were taken after 24 h incubation to minimize the role of cell proliferation in gap-filling and primarily to assess the ability of the cells to migrate. The analyses used a lower serum concentration (1% (*v*/*v*) FBS) in the culture medium (so-called serum starvation) [55]. Due to the fact that cell migration is an important step in the formation and repair of tissues, when assessing the therapeutic properties of plant extracts, the assessment of this migration can show the potential positive effect of given extracts in the process of tissue regeneration or wound healing. Scientific reports indicate that the proper processes of cell proliferation and migration play a very important role in maintaining good skin condition and proper healing of wounds [57]. The analyses carried out as part of this work to assess the effect of the *C. mas* L. extract and ferment on the migration of keratinocytes and fibroblasts showed that at a concentration of 1000 μg/mL, they stimulate cell proliferation during a 24-h incubation. The photo shows a slightly stronger promigratory effect of dogwood ferments than extracts. In the case of control cells not treated with the test samples, the migration process was much smaller, especially in the case of fibroblasts (Figure 9 and Figure 10).

### 2.6. Assessment of Antibacterial Activity

Various skin diseases are often accompanied by concomitant bacterial infections, which negatively affect the appearance and condition of the skin and significantly extend the time of treatment of skin diseases. Therefore, when assessing the potential use of plant extracts in cosmetic preparations, it is also extremely important to assess their antibacterial properties, especially against those bacteria that can contribute to the formation of skin lesions. The antibacterial properties of various types of dogwood extracts, from fruits, leaves and bark, have already been shown by other authors on various bacterial strains [36,58,59,60]. In this work, the research was extended with additional strains and the antibacterial properties between the extract and dogwood ferment were compared. Literature data indicate the antibacterial activity of *C. mas* L. against many bacteria such as *Staphylococcus aureus, Staphylococcus epidermidis, Pseudomonas aeruginosa, Streptococcus pyogenes, Clostridium perfringens, Bacillus cereus, Bacillus subtilis, Listeria monocytogenes, Sarcina lutea, Mariniluteicoccus flavus, Escherichia coli, Salmonella enteritidis, Shigella sonnei, Serratia marcescens, Klebsiella pneumoniae* and *Proteus vulgaris* [61,62,63,64,65]. Dogwood extracts also show antifungal activity, inhibiting the proliferation of *Candida albicans* and *Aspergillus fumigatus* [63,64,65]. Our study showed the possibility of inhibiting the growth of other microorganisms such as *Micrococcus luteus, Yersinia enterocolitica, Staphylococcus capitis* and *Corynebacterium xerosis.* The conducted analyses also showed differences between the activity of the extract and the ferment from *C. mas* L. The antibacterial activity was strictly dependent on the concentration of the tested samples, and in the case of most bacteria, a stronger effect was observed when the ferment was used (Table 3). The minimum inhibitory concentrations (MIC) values obtained during the analyses for the dogwood extract and ferment showed antibacterial properties in the tested concentration range (25–3000 µg/mL) against 6 of 7 tested strains for the extract and against all strains for the ferment. These results confirmed the stronger antibacterial properties of the ferment for which the MIC values were equal to or less than 100 ug/mL. Nevertheless, the values obtained for the extract also indicate its strong antibacterial effect (Table 4). The antibacterial effect of the analyzed extract and ferment was certainly related to the action of biologically active compounds present in both analyzed samples. As the chromatographic analyses show, the ferment contained larger amounts of the analyzed compounds, which is probably the reason for obtaining larger zones of bacterial growth inhibition. The extract and ferment from *C. mas* L. contain both phenolic compounds, iridoids and ellagitannins, which are compounds with antimicrobial activity proven in numerous studies [66,67,68].

So far, many mechanisms of antibacterial activity of plant extracts have been described. The antibacterial activity of extracts and chemical compounds of plant origin may be related to the impact on the synthesis and functions of key components of bacterial cells [69]. These may include destruction of cell membranes, disturbances in the biosynthesis of the cell wall and the production of various types of proteins, as well as inhibition of the synthesis of nucleic acids [70]. The antibacterial effect may also be associated with binding to peptidoglycan, phosphatidylglycerol and cardiolipin, which results in dissipation of proton movement and increased membrane permeability [71]. The synthesis of amino acids in bacterial cells may also be inhibited by inactivation of ribosomes [72]. Moreover, plant extracts can prevent or even inhibit the formation of bacterial biofilms, which indicates their potential use in the treatment of diseases closely related to the formation of such biofilms [73,74]. Moreover, studies by Nouska et al. also showed that extracts from *Cornus mas* L. promote the proliferation of probiotic microorganisms, such as *Lactobacillus paracasei* and *Lactobacillus plantarum*, which also indicates the possibility of using these extracts in cosmetic preparations with a probiotic effect and for the production of functional beverages [30].

## 3. Materials and Methods

### 3.1. Plant Material and Fermentation Procedure

The plant material was obtained from Dary Natury, a Polish company known for producing and distributing herbs in Grodzisk, Poland. The kombucha starter cultures were purchased from a commercial source in Poland. To obtain *C. mas* L. extracts, 15 g of fruit was mixed with 250 mL of sterile distilled water at room temperature (about 20 °C). The extraction process was conducted for 24 h on a magnetic stirrer. The obtained extracts were then filtered twice using Whatman filter paper No. 10 (Thermo Fisher Scientific, Göteborg, Sweden). The dogwood extract obtained in this way was used for the fermentation process, which was carried out based on the principles described by Antolak et al. [75]. To start fermentation, 25 g of sucrose was added to the extract, achieving a final concentration of 10% (m/v). Additionally, 5 g of SCOBY (Symbiotic Culture of Bacteria and Yeast) and 25 mL of kombucha starter were added to initiate the fermentation. Fermentation was carried out in previously sterilized glass beakers (1000 mL, height 18 cm, diameter 8 cm). The process was carried out for a period of 14 days at room temperature (about 22 °C). After the fermentation process, the resulting bioferment was filtered through sterile gauze. Then both the extract and the ferment were evaporated to dryness. Sterile distilled water was added to the dried samples to obtain stocks with a concentration of 100 mg/mL, which were then diluted to individual concentrations (depending on the analysis).

### 3.2. Determination of Biologically Active Compounds

All standards and reagents including MS grade formic acid, and MS grade acetonitrile were from Sigma-Aldrich (St. Louis, MO, USA). Deionized water was obtained from Ultrapure Millipore Direct-Q^®^ 3UV-R (Merck, KGaA, Taufkirchen, Germany).

An ultra-high performance liquid chromatographic system (UHPLC) Infnity Series II coupled with a DAD detector and an Agilent 6224 ESI/TOF mass detector (Agilent Technologies, Santa Clara, CA, USA) and an RP18 column Titan (Supelco, Sigma-Aldrich, Burlington, MA, USA) (10 cm × 2.1 mm i.d., 1.9 µm particle size) were used to achieve the separation [76]. A mobile phase consisting of water containing 0.05% formic acid (solvent A) and acetonitrile containing 0.05% formic acid (solvent B) was utilized at a flow rate of 0.2 mL/min. The gradient protocol employed was as follows: 0–8 min from 98% A to 93% A (from 2% to 7% B), 8–15 min from 93% A to 88% A (from 7% to 12% B), 15–29 min from 88% A to 85% A (from 12% to 15% B), 29–40 min from 85% A to 80% A (from 15% B to 20% B), and 40–60 min from 80% A to 65% A (from 20% B to 35% B). The thermostat was set to a temperature of 30 °C. DAD chromatograms were recorded from 200 to 600 nm. The MS-ESI settings included a drying gas temperature of 325 °C, a drying gas flow of 8 L/min, a nebulizer pressure of 30 psi, a capillary voltage of 3500 V, a skimmer voltage of 65 V, and fragmentor voltages of 180 V and 240 V. Negative mode ionization was used to acquire ions within the range of 100 to 1200 m/z. MS identification was conducted through comparison with standards or literature data in cases where standards were not available.

### 3.3. Determination of Antioxidant Properties

#### 3.3.1. DPPH (1,1-Diphenyl-2-picrylhydrazyl) Radical Scavenging Assay

The antioxidant properties of the tested extract and ferment obtained from *Cornus mas* L. were assessed using the DPPH radical assay described by Brand-Williams et al. [77]. During the analyses, the DPPH free radical scavenging of the tested samples was measured in the concentration range from 1 to 5000 µg/mL. Initially, 100 µL of test samples was applied to a 96-well plate, then 100 µL of 4 mM methanolic DPPH solution (Merck KGaA, Darmstadt, Germany) was added to each well, and the samples were thoroughly mixed. Trolox and ascorbic acid were used as positive controls, distilled water as negative control and extract/ferment without DPPH as blank. Then, the absorbance of the tested samples was measured at a wavelength of 517 nm. Measurements were taken every 5 min for 30 min using a UV-VIS Filter Max spectrophotometer (Thermo Fisher Scientific, Waltham, MA, USA). The analyses were carried out three times for each tested extract and ferment concentration. The antioxidant capacity was expressed as the percentage of DPPH radical scavenging using Equation (1). Then, the IC_50_ value was determined, which defines the concentration of the extract or ferment causing a 50% decrease in the initial concentration of the DPPH radical.

(1) $$\%\;DPPH\;scavenging\;=\;\frac{Abs\;control-Abs\;sample}{Abs\;control}\times 100$$

where: Abs sample—absorbance of the sample; Abs control—absorbance of the control sample.

#### 3.3.2. ABTS Scavenging Assay

To determine the antioxidant properties of *Cornus mas L.* extract and ferment, the ABTS^•+^ Scavenging Assay described by Miller et al. [78] was used. In the first step, the 7 mM ABTS solution (Merck KGaA, Darmstadt, Germany) and the 2.4 mM potassium persulfate solution were mixed in equal proportions. The solution prepared in this way was left for 14 h at room temperature (about 22°C). After this time, the solution was diluted with methanol to obtain an absorbance of about 1.0 at the wavelength of λ = 734 nm. Then, the dogwood extract and ferment (in the concentration range from 1 to 5000 µg/mL) were mixed with the ABTS solution and the absorbance of the prepared samples was measured at λ = 734 nm using a UV/VIS spectrophotometer (Thermo Fisher Scientific, Waltham, MA, USA). Trolox and ascorbic acid were used as positive controls, methanol as negative control and extract/ferment without ABTS as blank. Measurements were repeated three times for each tested sample. ABTS radical scavenging was calculated from the Equation (2). Then, the IC_50_ parameter was determined, which defines the concentration of the extract or ferment causing a 50% decrease in the initial concentration of the ABTS radical.

(2) $$\%\;of\;ABTS\;scavenging=1-\frac{Abs\;sample}{Abs\;control}\times 100$$

where: Abs sample—absorbance of the sample; Abs control—absorbance of the control sample.

#### 3.3.3. Fe^2+^ Chelation Assay

The antioxidant properties of *Cornus mas* L. were also determined by assessing the chelation of Fe^2+^ ions by the tested extract and ferment. Measurements were made in accordance with the methodology previously described by Gaweł-B̨eben et al. [79] with minor modifications. Initially, the dogwood extract and ferment (100, 250 and 1000 μg/mL) were mixed with distilled water, 1 mM FeCl_2_ (Merck KGaA, Darmstadt, Germany) and 5 mM ferrozine(Merck KGaA, Darmstadt, Germany). The negative control was ethanol, and the positive control was an ascorbic acid solution in analogous concentrations as in the case of the tested samples. The samples prepared in this way were thoroughly mixed and incubated at room temperature for 10 min. After this time, the absorbance of the test samples was measured at a wavelength of λ = 562 nm using an Aquamate Helion UV/VIS spectrophotometer (Thermo Fisher Scientific, Waltham, MA, USA). Three replicates were performed for each of the analyzed samples. The ability to chelate Fe^2+^ ions of the dogwood extract and ferment was calculated as the percentage of inhibition of the formation of the ferrozine-Fe^2+^ complex from the formula:

$$\%\;of\;Fe\;ions\;chelation=1-\frac{Abs\;sample}{Abs\;control}\times 100\;$$

where: Abs sample—absorbance od the sample; Abs control—absorbance of the control sample.

#### 3.3.4. Detection of Intracellular Levels of Reactive Oxygen Species (ROS)

In order to determine the ability of the *C.mas* L. extract and ferment to inhibit the intracellular production of reactive oxygen species in skin cells, analyses were performed based on the methodology previously described by Michalak et al. [80]. For this purpose, the fluorogenic H_2_DCFDA probe (Thermo Fisher Scientific, Waltham, MA, USA) was used. Initially, keratinocytes and fibroblasts were seeded separately in 96-well flat bottom plates at a density of 1 x 10^4^ cells/well. The cells on the plates were then grown in an incubator for 24 h to attach to the bottom of the wells. The DMEM (Dulbecco’s Modified Eagle Medium) medium was then removed from the wells and replaced with a solution of 10 μM H_2_DCFDA (Sigma Aldrich, Sant Louis, MO, USA) dissolved in DMEM medium without FBS serum. The cells (HaCaT and BJ) were then incubated in the dark with H_2_DCFDA for 45 min and then treated simultaneously with 500 μM H_2_O_2_ and test samples (extract or ferment) at concentrations of 100, 250 and 1000 μg/mL. Cells treated with 500 μM hydrogen peroxide (H_2_O_2,_ Merck KGaA, Darmstadt, Germany) were the positive control, while cells treated with neither test extracts nor H_2_O_2_ were control cells (both controls were prepared for both HaCaT and BJ cells). After 60 min of incubation, the fluorescence of 2′,7′-dichlorofluorescein (DCF) was measured in individual wells. Measurements were performed at an excitation wavelength of λ = 485 nm and an emission wavelength of λ = 530 nm using a microplate reader (FilterMax F5, Thermo Fisher Scientific, Waltham, MA, USA). As part of the analyses, three independent experiments were performed with each sample tested in triplicate.

### 3.4. Cytotoxicity Analysis

#### 3.4.1. Cell Culture

In order to determine the cytotoxicity of the analyzed dogwood extract and ferment, two types of skin cells were tested: fibroblasts (BJ, American Type Culture Collection Manassas, VA, USA) and keratinocytes (HaCaT, CLS Cell Lines Service GmbH, Eppelheim, Germany). Both cell types were maintained in DMEM (Dulbecco’s Modified Essential Medium, Biological Industries, Kibbutz Beit-Haemek, Israel) culture medium with L-glutamine supplemented with 10% (*v*/*v*) FBS (Fetal Bovine Serum, Merck KGaA, Darmstadt, Germany) and 1% (*v*/*v*) antibiotic (100 U/mL penicillin and 1000 µg/mL streptomycin, Merck KGaA, Darmstadt, Germany). Cells were grown in culture flasks in an incubator in a humidified atmosphere of 95% air and 5% carbon dioxide (CO_2_) at 37 °C. When the cells reached an appropriate confluence (approximately 70–80%), the culture medium was aspirated and the cells were rinsed twice with sterile PBS (Phosphate Buffered Saline, Biological Industries, Kibbutz Beit-Haemek, Israel). The cells were then trypsinized and placed in fresh DMEM medium. In the next step, the cells of fibroblasts and keratinocytes separately were transferred to a 96-well plate with a flat bottom at a density of 1 × 10^4^ cells/well and incubated for 24 h in an incubator. After this time, cells attached to the bottom were treated with individual concentrations of the *C. mas* L. extract and ferment.

#### 3.4.2. Alamar Blue Assay

The first test used to assess the viability of the examined skin cells was the Alamar Blue assay. This assay was performed according to the procedure described by Page et al. with modifications [81]. Briefly, analyses were performed on 96-well plates, on which individual skin cells (BJ and HaCaT) were seeded earlier. After a 24-h incubation of the cells with the test extract and ferment dissolved in DMEM medium at concentrations of 100, 250 and 1000 µg/mL, the samples were aspirated and resazurin solution (Merck KGaA, Darmstadt, Germany) at a concentration of 60 µM was added to each well. As a control, untreated cells cultured in DMEM medium were used. The plates were then placed in an incubator for 2 h at 37 °C. Fluorescence was then measured at λ = 570 nm using a FilterMax F5 microplate reader (Thermo Fisher Scientific, Waltham, MA, USA). As part of the analyses, three independent experiments were performed in which each sample was analyzed in triplicate.

#### 3.4.3. Neutral Red Uptake Assay

The second test used to assess the viability of skin cells was the Neutral Red (NR) uptake assay performed according to the procedure of Borrenfreund et al. with minor modifications [82]. After 24 h of incubation of BJ cells and HaCaT with the analyzed samples at concentrations of 100, 250 and 1000 µg/mL, a NR dye was added to each well in a 96-well plate at a concentration of 40 µg/mL. Plates prepared in this way were placed in an incubator for 2 h at 37 °C. The NR dye (Merck KGaA, Darmstadt, Germany) was then removed and the cells were washed with sterile PBS. After removing the PBS buffer, 150 µL of decolorizing buffer (C_2_H_5_OH/CH_3_COOH/H_2_O, 50%/1%/49%) was added to each well and absorbance measurements were performed at λ = 540 nm. As part of the analyses, three independent experiments were carried out, in which each sample was performed in triplicate.

#### 3.4.4. Fluorescence Live Cell Imaging

The effect of the analyzed extracts on the morphology of keratinocytes and fibroblasts was assessed with the use of two fluorophores: Hoechst33342 (Merck KGaA, Darmstadt, Germany) and calcein AM (Merck KGaA, Darmstadt, Germany). Hoechst33342 was used to stain the cell nucleus, and calcein AM to stain the cytoplasm of cells. Cell visualization was performed using an inverted fluorescence microscope (Carl Zeiss Microscopy GmbH, Biosciences, Jena, Germany) with excitation/emission peaks of 352 nm/454 nm (for Hoechst 33342) and 485 nm/530 nm (for calcein AM).

### 3.5. Evaluation of Matrix Metallopeptidases Inhibition

#### 3.5.1. Evaluation of Inhibition of Collagenase Activity

The ability to inhibit collagenase activity by *C. mas* L. extract and ferment was evaluated by fluorimetry using a commercially available kit (Abcam, Cambridge, UK, ab211108). Measurements were performed in accordance with the manufacturer’s instructions and the procedure previously described by Nizioł- Łukaszewska et al. [83]. Analyses were performed in black 96-well flat-bottomed plates for extract and ferment at concentrations of 100, 250 and 1000 µg/mL. First, collagenase (COL) was dissolved in collagenase analysis buffer (CAB). COL and CAB were then added to the wells. In addition, an inhibitor control sample was prepared by mixing 80 mM 1,10-phenanthroline (collagenase inhibitor) with collagenase and CAB. Diluted COL mixed with CAB was used as enzyme control and CAB buffer was used as a background control. Samples prepared in this way were then incubated at room temperature for 15 min. In the meantime, a reaction mixture was prepared by mixing the collagenase substrate with the CAB. After incubation, the reaction mixture was added to the wells and mixed thoroughly. Fluorescence measurements were performed at the excitation wavelength λ = 490 nm and the emission wavelength λ = 520 nm. Measurements were performed in kinetic mode for 60 min at 37 °C. The ability of individual extract and ferment concentrations to inhibit collagenase activity was calculated using Equation (3):

(3) $$\%\;relative\;COL\;inhibition=\frac{enzyme\;control-sample}{enzyme\;control}\times 100$$

#### 3.5.2. Evaluation of Inhibition of Elastase Activity

The ability to inhibit collagenase activity by *C. mas* L. extract and ferment was evaluated by fluorimetry using a commercially available kit (ab118971, Abcam, Cambridge, UK). The evaluation was carried out in accordance with the manufacturer’s instructions and the procedure previously described by Nizioł-Łukaszewska et al. [83]. Briefly, extract and ferment at concentrations of 100, 250 and 1000 µg/mL were placed in a 96-well black flat-bottomed plate. Neutrophil elastase (NE), elastase substrate and control inhibitor (SPCK) solutions were then prepared according to the manufacturer’s instructions. The NE solution was diluted as appropriate and added to the individual wells. Inhibitor control and enzyme control (assay buffer) were added to other wells instead of test samples. The contents of the wells were thoroughly mixed and then incubated at 37 °C for 5 min. In the meantime, a reaction mixture was prepared by mixing the assay buffer with the NE substrate. After incubation, the reaction mixture was added to the wells and mixed thoroughly. Fluorescence measurements were started immediately with a microplate reader (FilterMax F5, Thermo Fisher Scientific, Waltham, MA, USA) at excitation wavelength λ = 400 nm and emission wavelength λ = 505 nm. The measurements were performed in kinetic mode for 40 min at 37 °C. The ability of the analyzed samples to inhibit NE activity was calculated using Equation (4):

(4) $$\%\;relative\;NE\;activity=\frac{\Delta RFU\;test\;inhibitor}{\Delta RFU\;enzyme\;control}\times 100$$

### 3.6. Scratch Wound Assay

In order to assess the possibility of stimulating the migration of keratinocytes and fibroblasts by the dogwood extract and ferment, a scratch test was performed based on the procedure described earlier by Nizioł-Łukaszewska et al. [83]. This assay was carried out in 6-well flat bottom plates. After the cell monolayer (both HaCaT and BJ) reached the appropriate confluence, the cell layer was carefully scraped off with a 10-μL pipette tip. Then, cells in individual wells were treated with *C. mas* L. extract and ferment at concentrations of 1000 µg/mL. Tested samples were dissolved in DMEM culture medium with reduced FBS content (1% (*v*/*v*)). After 24 h of incubation of the cells with the tested samples, photos of the cells with the previously made scratch were taken under an inverted microscope at 10x magnification (Nikon Eclipse TS 100-F, Nikon, Warsaw, Poland).

### 3.7. Assessment of Antibacterial Activity

#### 3.7.1. Disk-Diffusion Assay

The disk-diffusion method was used to assess the effect of the analyzed extract and ferment from *Cornus mas* L. on the growth of pathogenic bacteria. The analyzed strains were obtained from the American Type Culture Collection (Manassas, VA, USA). The organisms used in the tests were *Staphylococcus aureus* ATCC BAA-2312, *Staphylococcus epidermidis* ATCC^®^ 49134™, *Staphylococcus capitis* ATCC^®^ 146™, *Micrococcus luteus* ATCC^®^ 10240™, *Corynebacterium xerosis* ATCC^®^ 373, *Yersinia enterocolitica* ATCC 27729 and *Pseudomonas aeruginosa* ATCC^®^ 35032. To assess the zone of growth inhibition, 10 mL of agar medium appropriate for each bacterial strain was poured onto sterile Petri dishes. For this purpose, Tryptone Soy Agar, Mannitol Salt Agar, Mueller Hinton Agar, Nutrient Agar, Tryptone Soya Blood Agar, Blood Agar, LB Agar and MRS Agar were used. All bacterial media were purchased from Argenta (Poznan, Poland). Then, the media were inoculated with individual strains of microorganisms with a density of 5 × 10^7^ CFU (colony-forming unit)/mL. Then, individual dilutions (100 and 1000 µg/mL) of the extract and ferment were prepared and sterilized through membrane filters (0.22 µm). Sterile filter paper discs (6 mm in diameter) were soaked in the tested concentrations of the extract and ferment, and then the discs were placed on the surface of the solidified agar medium using sterile tweezers. A disc soaked in sterile distilled water was used as a negative control. Appropriate antibiotics inhibiting the growth of the tested strains were used as a positive control. The seeded plates with the impregnated discs were left at 4 °C for 2 h to allow the compounds to pre-diffuse in the medium, then the plates were transferred to an incubator and grown at 35 ± 2 °C. The influence of the tested samples on the growth of individual strains of microorganisms was assessed by measuring the diameter of the inhibition zone after 24 h of culture.

#### 3.7.2. Determination of Minimum Inhibitory Concentrations (MIC)

The antibacterial activity of the *C. mas* L. extract and ferment against the above-mentioned bacterial strains was also assessed by determining the minimum inhibitory concentration (MIC). For this purpose, the broth microdilution technique with p-iodonitrotetrazolium violet (INT, Merck KGaA, Darmstadt, Germany) as a growth indicator, which was described by Eloff [84], was used. The extract and ferment were serially diluted using broth to give concentrations ranging from 25 to 3000 µg/mL. Tested samples in this range of concentrations were placed in 96-well microplates. A suspension of each bacterial strain containing 5 × 10^4^ colony-forming units was then added to each well. A sterility control well, an appropriate antibiotic well and a growth control well were also prepared for each bacterial strain tested. The prepared plates were incubated at 37 °C for 24 h, after which 40 μL of 0.4 mg/mL INT solution was added to the wells. The plates were incubated again at 37 °C for a further 30 min. The concentration of the extract or ferment that inhibited the total growth of the tested bacterial strains was taken as the MIC value. As part of the analysis, three independent experiments were performed.

### 3.8. Statistical Analysis

The values of various parameters measured during the performed analyses were expressed as mean ± standard deviation (SD). A two-way analysis of variance (ANOVA) and a post hoc intergroup Dunnett’s test were performed. Statistical significance was determined at **** *p* < 0.0001, *** *p* < 0.001, ** *p* < 0.01 and * *p* < 0.05 compared to control. Statistical analyses of the obtained results were performed using GraphPad Prism 8.4.3 (GraphPad Software, Inc., San Diego, CA, USA).

## 4. Conclusions

The results of the analyses carried out as part of this work indicate the beneficial properties of both the extract and the ferment from *Cornus mas* L. and their potential use in preparations for the care and treatment of skin diseases. The obtained results indicate that the ferment obtained after the fermentation of the dogwood extract is characterized by a higher content of biologically active compounds and has stronger antioxidant properties compared to the extract. What is more, the dogwood bioferment obtained with a SCOBY has a more stimulating effect than the extract on the viability of both fibroblasts and keratinocytes and increases the metabolic activity and proliferation of these skin cells. Moreover, the extracts and ferments have no adverse effect on cell morphology and can stimulate the migration of both types of skin cells in vitro. In addition, both the extract and the ferment show the ability to inhibit the activity of elastase and collagenase, which may contribute to delaying the aging process of the skin. The antibacterial properties of the tested samples are also promising, indicating the possibility of using both the dogwood extract and the ferment in preparations intended to combat skin diseases accompanied by bacterial infections. To sum up, this paper proposed for the first time the use of *Cornus mas* L. as an alternative raw material to *Camellia sinensis* for fermentation with kombucha. It has also been shown that in addition to the various biological activities proven in numerous studies, this plant may have an anti-aging effect by delaying the degradation processes of collagen and elastin. What is more, the work indicates a stronger effect of the ferment from *Cornus mas* L. than the extract, which shows the producers of cosmetic or pharmaceutical preparations another method of obtaining biological samples with a wide spectrum of activity and indicates the legitimacy of carrying out fermentation processes of various plant raw materials. In connection with obtaining promising results in the context of the potential use of dogwood in cosmetology and dermatology, our research in the near future will be based on an extended assessment of the properties of cosmetic products based on dogwood extracts and ferments and the assessment of their anti-inflammatory, anti-hyperpigmentation and moisturizing properties, as well as determination of their cytotoxicity against skin cancer cells.

## Figures and Tables

**Table 1:** Mass data and results of quantitative analysis of polyphenols and irydoids (mean ± SD) in extract and ferment from fruit of *C. mas* L.

Rt (min)	Observed Ion Mass [M-H]-/(Fragments)	Δ ppm	Formula	Identified	Extract (µg/g)	Ferment (µg/g)
1.44	195.05164	3.13	C_6_H_12_O_7_	Gluconic acid *	+	++
1.54	191.05677	3.43	C_7_H_12_O_6_	Quinic acid *	+	++
2.99	331.06841 (125,169)	4.03	C_13_H_16_O_10_	O-galloyl-d-glucose ^1^	30.2 ± 1.2	47.7 ± 2.3
3.44	169.01501 (125)	4.49	C_7_H_6_O_5_	Gallic acid *	95.5 ± 5.2	181.1 ± 9.5
4.26	361.07901/(125,169)	3.81	C_14_H_18_O_11_	Galloyl-d-sedoheptulose ^1^	8.0 ± 0.3	18.0 ± 1.1
5.87	315.07298 (153)	2.61	C_13_H_16_O_9_	Dihydroxybenzoic acid glucoside	+	+
6.65	153.01989	3.62	C_7_H_6_O_4_	Protocatechuic acid *	15.1 ± 1.1	13.3 ± 1.1
12.19	375.13001	0.90	C_16_H_24_O_10_	Loganic acid *	180.0 ± 9.7	556.1 ± 24.4
13.70	447.09411 (285)	1.84	C_21_H_20_O_11_	Cyanidin 3-O-galactoside *	++	+
14.29	353.08856 (191,179)	2.13	C_16_H_18_O_9_	Chlorogenic *	-	+
14.97	491.14121 (375)	1.18	C_20_H_28_O_14_	Loganic acid derivative ^2^	15.1 ± 1.1	40.0 ± 3.1
16.32	491.14187 (375)	2.52	C_20_H_28_O_14_	Loganic acid derivative ^2^	-	6.1 ± 0.3
16.80	403.12521	1.55	C_17_H_24_O_11_	Secoxyloganin ^2^	16.2 ± 3.1	60.1 ± 3.2
35.02	541.15699	1.31	C_24_H_30_O_14_	Cornuside *	5.2 ± 0.0	52.2 ± 3.5
43.41	723.50621	1.29	C_41_H_72_O_10_	unidentified	+	++
44.77	836.58703	0.45	C_44_H_85_O_14_	unidentified	-	++
48.49	836.58724	0.70	C_44_H_85_O_14_	unidentified	+	++

* Identification was confirmed using standard; ^1^—quantification was carried out based on calibration curve for gallic acid; ^2^—quantification was carried out based on calibration curve for loganic acid; - not detected; + detected; ++ present at high concentration.

**Table 2:** DPPH and ABTS radical scavenging IC50 values for *Cornus mas* L. extract and ferment. Values are means ± standard deviation (SD) of triplicates.

	Extract	Ferment
DPPH Assay		
IC_50_ [µg/mL]	100.2 ± 2.34 **	99.7 ± 1.56 **
ABTS Assay		
IC_50_ [µg/mL]	92 ± 0.95	91 ± 0.72

** denotes significant differences between the values, with *p* = 0.0078.

**Table 3:** Antibacterial activity of tested extract and ferment expressed as the diameter of the average inhibition zone (mm).

Bacteria	Zone of Inhibition [mm]			
	Extract		Ferment	
	100 µg/mL	1000 µg/mL	100 µg/mL	1000 µg/mL
*Staphylococcus aureus*	nd	nd	8	9
*Staphylococcus epidermidis*	nd	12	8	10
*Staphylococcus capitis*	nd	nd	8	8
*Micrococcus luteus*	10	7	11	9
*Corynebacterium xerosis*	7	9	10	11
*Yersinia enterocolitica*	9	10	14	10
*Pseudomonas aeruginosa*	7	7	9	12

nd—not detected.

**Table 4:** Minimum inhibitory concentrations (MIC) of *Cornus mas* L. against the tested bacteria.

Bacteria	Minimum Inhibitory Concentration MIC [µg/mL]	
	Extract	Ferment
*Staphylococcus aureus*	1500	100
*Staphylococcus epidermidis*	250	50
*Staphylococcus capitis*	nd	100
*Micrococcus luteus*	50	25
*Corynebacterium xerosis*	100	25
*Yersinia enterocolitica*	50	25
*Pseudomonas aeruginosa*	50	25

nd—not detected.

## Data Availability

The data presented in this study are available on request from the corresponding author.

## Supplementary Materials

The following supporting information can be downloaded at: https://www.mdpi.com/article/10.3390/ijms241713232/s1.
