# Supplementary material for: Comparative Study of Cytotoxicity and Antioxidant, Anti-Aging and Antibacterial Properties of Unfermented and Fermented Extract of Cornus mas L
Source: Int J Mol Sci. 2023 Aug 26;24(17):13232. doi: 10.3390/ijms241713232 (PMC10487488; doi:10.3390/ijms241713232)
Supplement: Supplementary file 1 [file ijms-24-13232-s001.zip › ijms-2556649-supplementary.pdf]

International Journal of *Molecular Sciences*

MDPI

Article

Comparative Study of Cytotoxicity and Antioxidant, Anti-Aging and Antibacterial Properties of Unfermented and Fermented Extract of *Cornus mas* L.

Martyna Zagórska-Dziok [1], Aleksandra Ziemlewska [1], Agnieszka Mokrzyńska [1], Zofia Nizioł-Łukaszewska [1], Ireneusz Sowa [2], Dariusz Szczepanek [3] and Magdalena Wójciak [2,*]

[1] Department of Technology of Cosmetic and Pharmaceutical Products, Medical College, University of Information Technology and Management in Rzeszow, Sucharskiego 2, 35-225 Rzeszow, Poland; mzagorska@wsiz.edu.pl (M.Z.-D.); aziemlewska@wsiz.edu.pl (A.Z.); amokrzynska@wsiz.edu.pl (A.M.); zniziol@wsiz.edu.pl (Z.N.-Ł.)

[2] Department of Analytical Chemistry, Medical University of Lublin, Aleje Raclawickie 1, 20-059 Lublin, Poland; i.sowa@umlub.pl

[3] Chair and Department of Neurosurgery and Pediatric Neurosurgery, Medical University of Lublin, Jaczewskiego 8, 20-090 Lublin, Poland; dariusz.szczepanek@umlub.pl

* Correspondence: magdalena.wojciak@umlub.pl

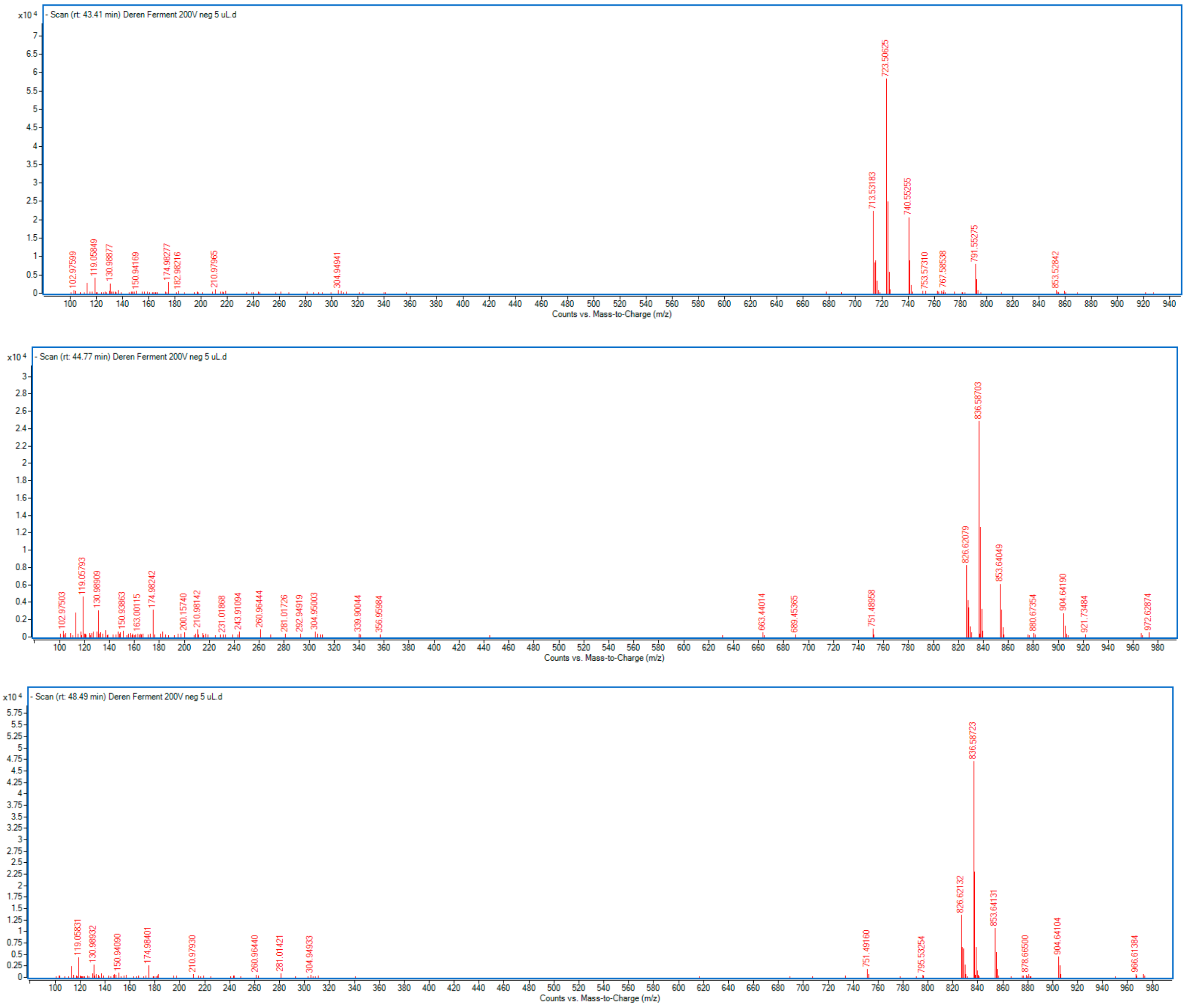

Figure S1. Representative MS spectra of unknown components found in *C. mas* fruit extract. MS data are given in Table 1.

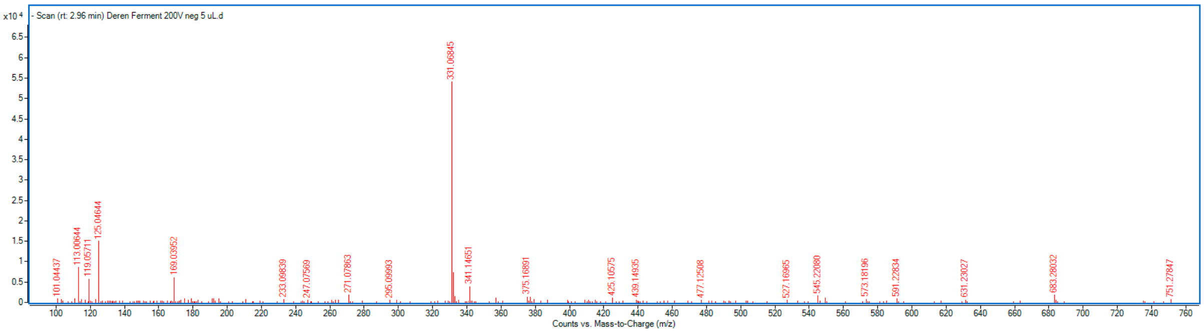

x10 4
- Scan (rt: 2.96 min) Deren Ferment 200V neg 5 uL.d
331.06845
101.04437
113.00644
119.05711
125.04644
169.03952
233.09839
247.07569
271.07863
295.09993
341.14651
375.16891
425.10575
439.14935
477.12508
527.16965
545.22080
573.18196
591.22834
631.23027
683.28032
751.27847
Counts vs. Mass-to-Charge (m/z)

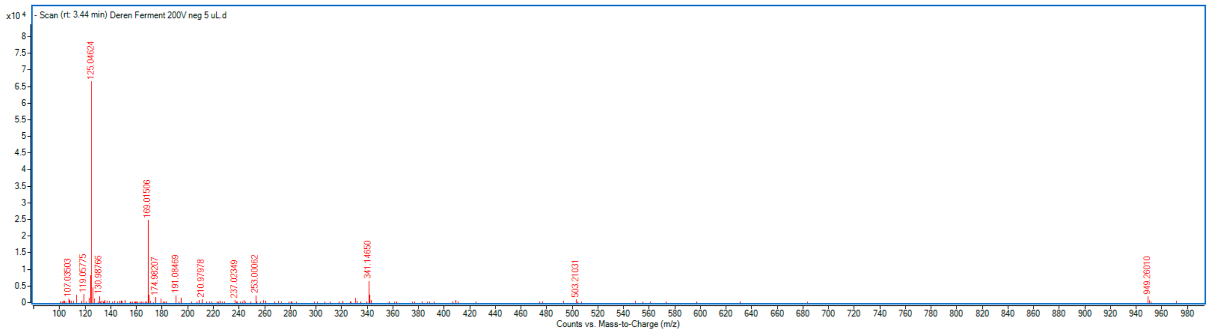

x10 4
- Scan (rt: 3.44 min) Deren Ferment 200V neg 5 uL.d
125.04624
169.01506
107.03503
119.05775
130.98766
174.98207
191.08469
210.97978
237.02349
253.00062
341.14650
503.21031
949.26010
Counts vs. Mass-to-Charge (m/z)

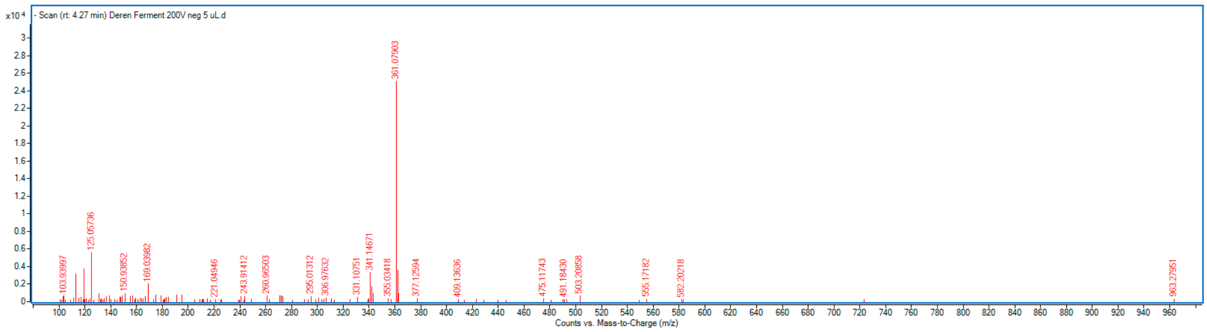

x10 4
- Scan (rt: 4.27 min) Deren Ferment 200V neg 5 uL.d
361.07903
125.05736
103.93997
150.93852
169.03982
221.04946
243.91412
260.96503
295.01312
306.97632
331.10751
341.14671
355.03418
377.12594
409.13636
475.11743
491.18430
503.20858
555.17182
582.20218
963.27951
Counts vs. Mass-to-Charge (m/z)

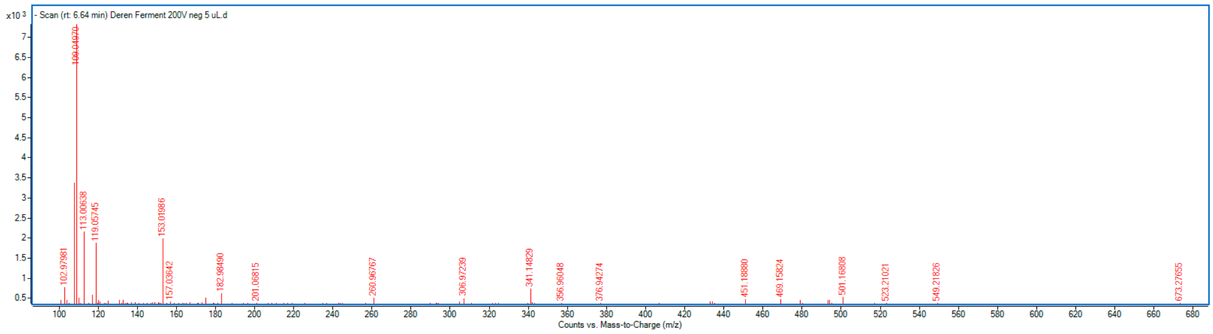

x10 3
- Scan (rt: 6.64 min) Deren Ferment 200V neg 5 uL.d
109.04970
102.97981
113.00638
119.05745
153.01986
157.03642
182.98490
201.06815
260.96767
306.97239
341.14829
356.96048
376.94274
451.18880
469.15824
501.16808
523.21021
549.21826
673.27955
Counts vs. Mass-to-Charge (m/z)

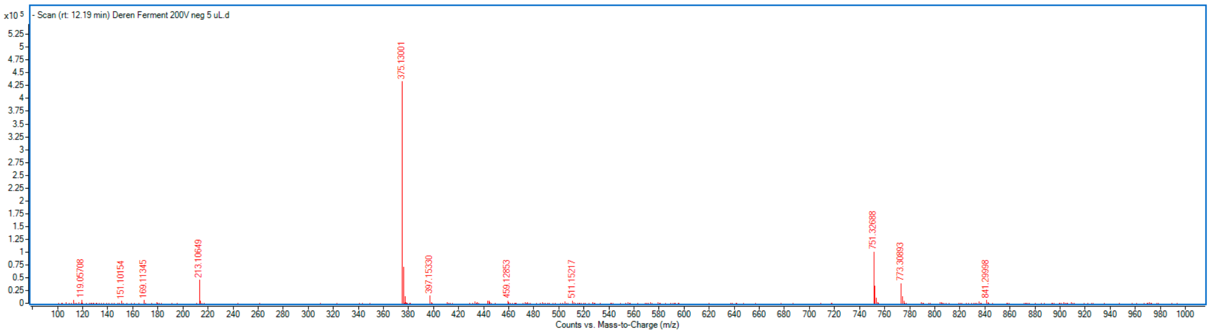

x10 5
- Scan (rt: 12.19 min) Deren Ferment 200V neg 5 uL.d
375.13001
119.05708
151.10154
169.11345
213.10649
397.15330
459.12853
511.15217
751.32688
773.30893
841.29998
Counts vs. Mass-to-Charge (m/z)

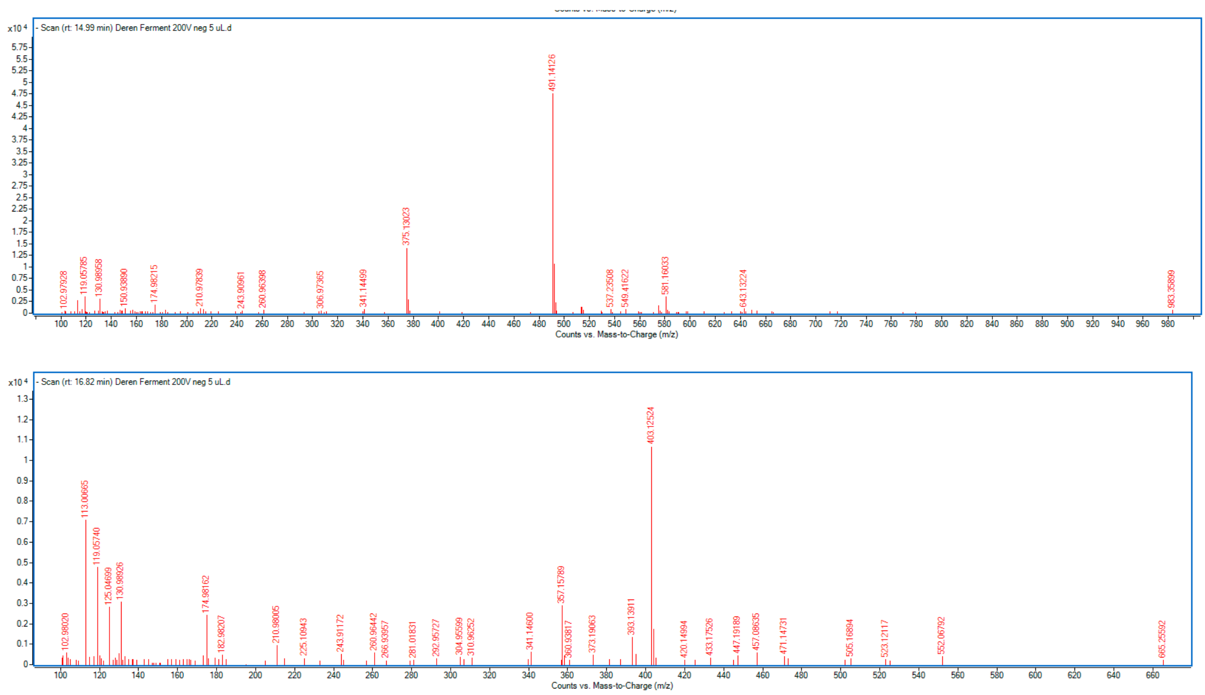

Figure S2. Representative MS spectra of main identified components found in *C. mas* fruit extract. MS data are given in Table 1.
